# A weighted cranial diffusion-weighted imaging scale for Wilson’s disease

**DOI:** 10.3389/fnins.2023.1186053

**Published:** 2023-08-15

**Authors:** Shi-jing Wang, Hao Geng, Si-rui Cheng, Chen-chen Xu, Rui-qi Zhang, Yu Wang, Tong Wu, Bo Li, Tao Wang, Yong-sheng Han, Zeng-hui Ding, Yi-ning Sun, Xun Wang, Yong-zhu Han, Nan Cheng

**Affiliations:** ^1^Graduate School, Anhui University of Chinese Medicine, Hefei, China; ^2^Hospital Affiliated to the Institute of Neurology, Anhui University of Chinese Medicine, Hefei, China; ^3^Institute of Intelligent Machines, Hefei Institute of Physical Science, Chinese Academy of Sciences, Hefei, China; ^4^Department of Biophysics, University of Science and Technology of China, Hefei, China; ^5^Department of Economics, Nankai University, Tainjin, China

**Keywords:** Wilson disease, rating scores, MRI, neuroimaging, clinical assessment

## Abstract

**Objectives:**

Cranial magnetic resonance imaging (MRI) could be a crucial tool for the assessment for neurological symptoms in patients with Wilson’s disease (WD). Diffusion-weighted imaging (DWI) hyperintensity reflects the acute brain injuries, which mainly occur in specific brain regions. Therefore, this study aimed to develop a weighted cranial DWI scale for patients with WD, with special focus on specific brain regions.

**Materials and methods:**

In total, 123 patients with WD were enrolled, 118 of whom underwent 1.5 T-MRI on admission. The imaging score was calculated as described previously and depended on the following sequences: one point was acquired when abnormal intensity occurred in the T1, T2, and fluid-attenuation inversion recovery sequences, and two points were acquired when DWI hyperintensity were found. Consensus weighting was conducted based on the symptoms and response to treatment.

**Results:**

Intra-rater agreement were good (*r* = 0.855 [0.798–0.897], *p* < 0.0001). DWI hyperintensity in the putamen was a high-risk factor for deterioration during de-copper therapy (OR = 8.656, *p* < 0.05). The high-risk factors for readmission for intravenous de-copper therapies were DWI hyperintensity in the midbrain (OR = 3.818, *p* < 0.05) and the corpus callosum (OR = 2.654, *p* < 0.05). Both scoring systems had positive correlation with UWDRS scale (original semi-quantitative scoring system, *r* = 0.35, *p* < 0.001; consensus semi-quantitative scoring system, *r* = 0.351, *p* < 0.001.). Compared to the original scoring system, the consensus scoring system had higher correlations with the occurrence of deterioration (OR = 1.052, 95%CI [1.003, 1.0103], *p* < 0.05) and readmission for intravenous de-copper therapy (OR = 1.043, 95%CI [1.001, 1.086], *p* < 0.05).

**Conclusion:**

The predictive performance of the consensus semi-quantitative scoring system for cranial MRI was improved to guide medication, healthcare management, and prognosis prediction in patients with WD. For every point increase in the neuroimaging score, the risk of exacerbations during treatment increased by 5.2%, and the risk of readmission to the hospital within 6 months increased by 4.3%.

## 1. Introduction

Wilson’s disease (WD) is an autosomal recessive copper metabolism disorder caused by mutations in the *ATP7B gene* (Członkowska et al., 2018). According to the main symptoms and organs with copper deposits, patients with WD can be divided into hepatic, neurological, and hepatic-neurological subtype (Cheng et al., 2017). Copper-chelating therapy has been used as the main treatment for most patients with WD and is effective in patients with the hepatic subtype, especially in the early-stages of the disease course (Walshe and Yealland, 1993). However, symptom worsening could occur in neurological subtype and hepatic-neurological subtype WD patients during early stage by copper-chelating treatment (Cheng et al., 2014). Currently, this deterioration is predicted through estimation by experienced physicians, instead of reliable biomarkers. Therefore, precise prediction, rather than empirical judgment, of the deterioration in neurological symptoms and prognosis has become a crucial target in recent therapeutic research on WD.

Neurological symptoms are caused by long-term copper accumulation in different brain regions (Masełbas et al., 2010; Hefter et al., 2018). Injuries can be found in putamen, globus pallidum, caudate nucleus, inner capsule, thalamus, midbrain, pons, medulla, cerebellum, cortex, and corpus callosum (Yu et al., 2019). The injury mode can be referred into demyelination, microglial activation, central pontine myelinolysis, and injury of gray/white matter (Dusek et al., 2017). Brain imaging can directly monitor the lesion, presenting as atrophy (Smolinski et al., 2019), white matter hyperintensity, increased magnetic susceptibility (Yang et al., 2015) and diffusion disorders (Favrole et al., 2006). Dusek developed a semi-quantitative MRI scale for patients with WD (Dusek et al., 2020; Rędzia-Ogrodnik et al., 2022). They paid special attention to sequences of T1, T2, Flair and SWI. Acute toxicity scores, chronic damage scores and atrophy scores were determined by the occurrence of T2/FLAIR hyperintensity, T2/T2*/SWI hypointensity and T1, respectively. WD cranial MRI severity was defined by acute injury and chronic damage (including atrophy) simultaneously, semi-quantified (Normal/absent = 0, mild/moderate = 1, severe = 2) to a total score. Intrarater and interrater agreement of the scale were analyzed, and it was verified as a reliable instrument. Meanwhile, the association between clinical and imaging severity proved that it was an effective tool to assess the severity of patients with WD.

DWI hyperintensity showed a higher specificity and clinical predictive value than other sequences. Favrole et al. (2006) found that hyperintense lesions were detected in all symptomatic patients on FLAIR MR images but only in 11 of 13 patients with WD who had typical neurological manifestations on DWI. Additionally, the reduction in diffusion changes was correlated to clinical improvement in patients with WD. Furthermore, lesions intensively occurred in specific brain regions rather than the whole brain among patients with WD. There was a corresponding relationship between different brain region injuries and different symptoms. Thalamic injury, brainstem/cerebellar injury, and corpus callosum injury were associated with a longer disease course, ataxia, and neuropsychiatric symptoms, respectively (Zhong et al., 2019; Zhou et al., 2019). Therefore, DWI hyperintensity in specific brain regions should be taken into crucial consideration as WD cranial MRI severity assessment.

Based on the semi-quantitative MRI scale for patients with WD, we aimed to weighting the specific brain regions according to the DWI hyperintensity and add it to the total WD cranial MRI severity score. Firstly, analysis was conducted to establish a corresponding correlation between injuries in specific brain regions and clinical symptoms, brain regions of interest and weighting coefficients were determined by data calculation and literature analysis. Secondly, weighting diffusion-weighted imaging (DWI) hyperintensity scores were added into WD cranial MRI severity scores. The weighted scores were then analyzed in relation to clinical symptoms, quality of life, and biomarkers. Thirdly, analyzing the relationship between weighted scoring and the worsening of symptoms during treatment, as well as the likelihood of readmission within 6 months or 12 months, to evaluate its clinical predictive value. We hope that this study will further strengthen MRI as an adjunctive examination to evaluate WD and can also become an important marker for predicting the prognosis of WD.

## 2. Materials and methods

### 2.1. Participants and general information

This cohort study was performed at the Center of WD, the affiliated hospital of the Institute of Neurology, Anhui University of Chinese Medicine. The cranial imaging analysis was conducted and confirmed by the Hefei Institute of Physical Science, Chinese Academy of Sciences. The study protocol was approved by the Ethics Committee of the Hefei Institutes of Physical Science, Chinese Academy of Sciences (SWYX-Y-2021-08). Written informed consent was obtained from all subjects by the principal researcher after a self-motivated behavior evaluation of the patient’s capacity to provide consent. The consent form was signed by the parents of patients under 18 years old.

At baseline, we enrolled 118 inpatients admitted between June 2019 and June 2020 with a diagnosis of WD, as per the Chinese guidelines for the diagnosis and treatment of WD in 2021 (Inherited Metabolic Liver Disease Collaboration Group, Chinese Society of Hepatology, Chinese Medical Association, 2022). WD should be considered in patients with unexplained liver disease, neurological symptoms (especially extrapyramidal symptoms), or psychiatric symptoms. Age of onset cannot be used as a basis for diagnosing or ruling out WD. The recommended diagnostic criteria include: (1) Neurological and/or psychiatric symptoms. (2) Unexplained liver damage. (3) Decreased serum ceruloplasmin and/or elevated 24-h urine copper (strong recommendation, moderate-quality evidence). (4) Positive Kayser-Fleischer (K-F) rings (strong recommendation, moderate-quality evidence). (5) Identification of pathogenic mutations in both chromosomes carrying the ATP7B gene through pedigree segregation and genetic analysis (strong recommendation, moderate-quality evidence). WD can be confirmed when meeting either (1 or 2) plus (3 and 4), or (1 or 2) plus 5. Individuals with criteria 3 plus 4 or 5 but without obvious clinical symptoms are diagnosed as pre-symptomatic individuals. Individuals meeting any two of the first three criteria are diagnosed as “possibly WD, “and further follow-up observation is recommended, with ATP7B gene testing suggested for a definitive diagnosis. The Chinese guidelines for WD emphasize early and lifelong treatment, as well as lifelong monitoring. For patients in the pre-symptomatic stage, zinc preparations can be used for maintenance therapy. Patients already receiving copper-chelating therapy should be monitored for indicators such as complete blood count, liver function, 24-h urine copper, and cranial MRI. The guidelines also provide recommendations for dietary control, including fasting, moderate intake, appropriate consumption, and recommended foods. There are differences in the use of copper-chelating agents between the Chinese guidelines and other guidelines. In China, sodium Dimercaptosulphonate (DMPS), Dimercaptosuccinic acid (DMSA) capsules, zinc gluconate, and traditional Chinese medicine are specifically considered. Among these, DMPS has a higher priority in copper-removing therapy for WD compared to penicillamine. DMSA and zinc gluconate offer alternative options for maintenance therapy and are suitable for patients allergic to penicillamine or with leukopenia. Trientine is not used in China for specific reasons.

**Figure 1:**
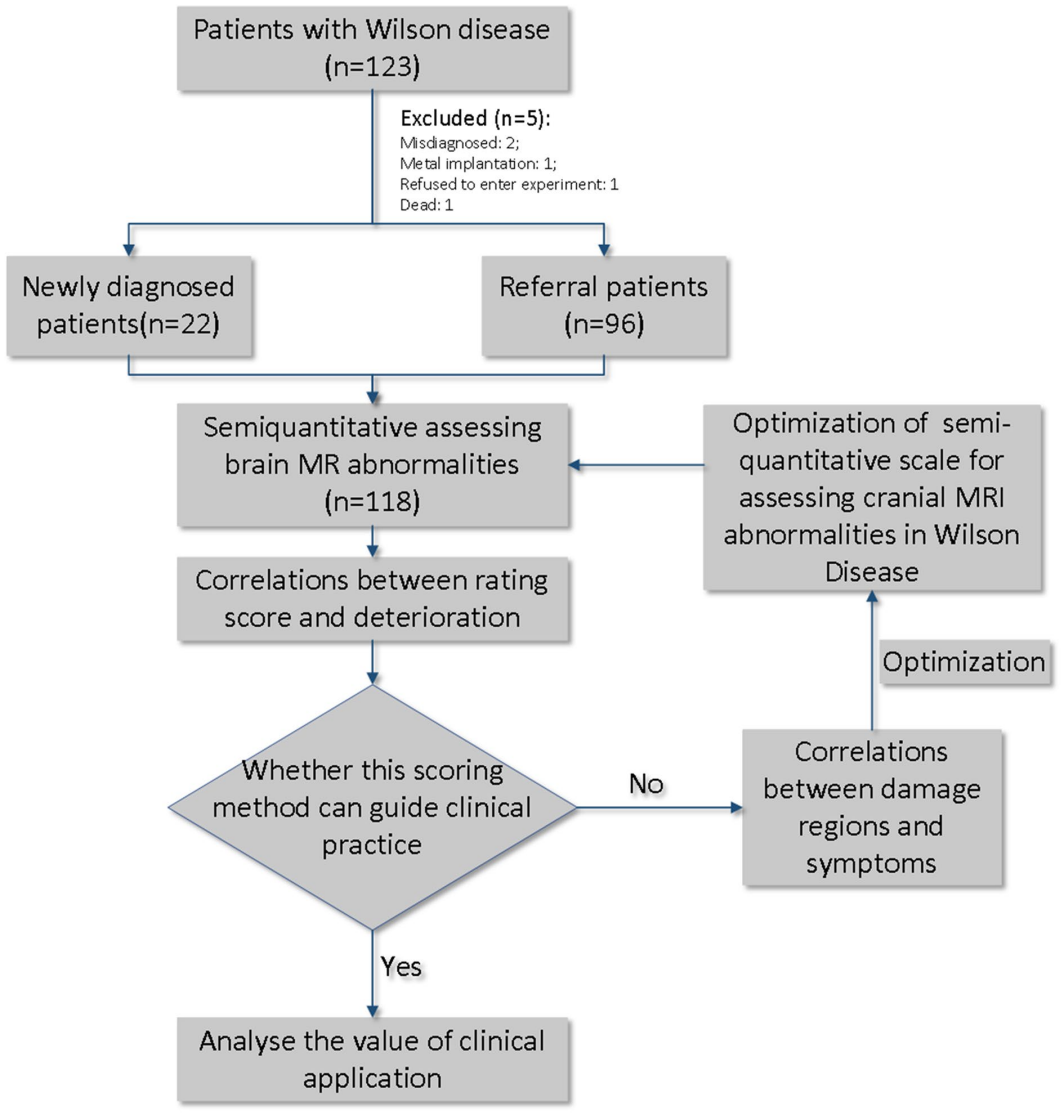
General idea and flow chart of this study.

Among these, 96 patients had been previously diagnosed with WD (referral patients), while 22 were newly diagnosed. At the time of admission, the Unified Wilson Disease Rating Scale (UWDRS) and cranial MRI examinations were conducted to assess the state of the disease. Patients were evaluated for the presence of any neurologic findings by 2 neurologists (R.W., 5 years of experience; XP.W., >30 years of experience). All patients received Cu-chelating therapy according to the Chinese guidelines for the diagnosis and treatment of WD (Neurogenetics Group and Neurology Branch of Chinese Medical Association, 2021). During the course of treatment, serum copper, ceruloplasmin, and urine copper at 24 h before treatment and the highest urine copper at 24 h during treatment were tested to determine the efficacy of treatment (Figure 1).

### 2.2. Parameters of magnetic resonance

Philips Achieva 1.5 T MRI scanning equipment was used to scan the images of patients with WD. The scans ranged from the foramen magnum to the upper edge of the corpus callosum. The scan sequences included T1WI, T2WI, FLAIR, and DWI. The scanning parameters were FLAIR, TR/TE = 9,000 ms/140 ms, flip angle 120°, slices thickness 6.5 mm, slices spacing 1.3 mm; DWI (b = 0, 1,000 s/mm^2^), single excitation SE-EPI sequence, TR/TE = 2,400 ms/104 ms, FOV 220 mm × 220 mm, matrix 168 × 105, slice thickness 6.5 mm, slice spacing 1.3 mm, number of slices 17 ~ 18 slices.

### 2.3. Image-scoring method and determination of weight coefficients

The image score evaluations were performed by two radiologists. According to previous studies (Favrole et al., 2006; Sinha et al., 2006), T1, T2, FLAIR, and DWI sequences were selected as reference sequences. The regions of interest included the caudate nucleus, putamen, globus pallidus, internal capsule, thalamus, midbrain, pons, cerebellum, medulla oblongata, cortex, and corpus callosum. T2/FLAIR hyperintensity was scored as 1 point, T1 abnormal signal intensity was scored as 1 point, and DWI hyperintensity was scored as 2 points. Subsequently, we conducted logistic regression analysis, using specific brain region imaging scores as independent variables, and the presence of clinical symptoms and prognostic indicators as dependent variables. Clinical symptoms consisted of dysarthria, dysphagia, gait disturbances, dystonia, muscular hypertonia, and psychiatric disorders. Prognostic indicators included post-treatment deterioration and readmission within 6–12 months. We identified statistically significant data and extracted the corresponding brain regions along with their corresponding odds ratios (OR). Weight coefficients were based on brain regions and corresponding OR mainly, as well as experts’ experience, literature reviews and actual application. For example, midbrain lesions are rare and worthless compared to corpus callosum lesions and caudate nucleus lesions. Therefore, the weight assigned to the midbrain region is reduced accordingly.

**Figure 2:**
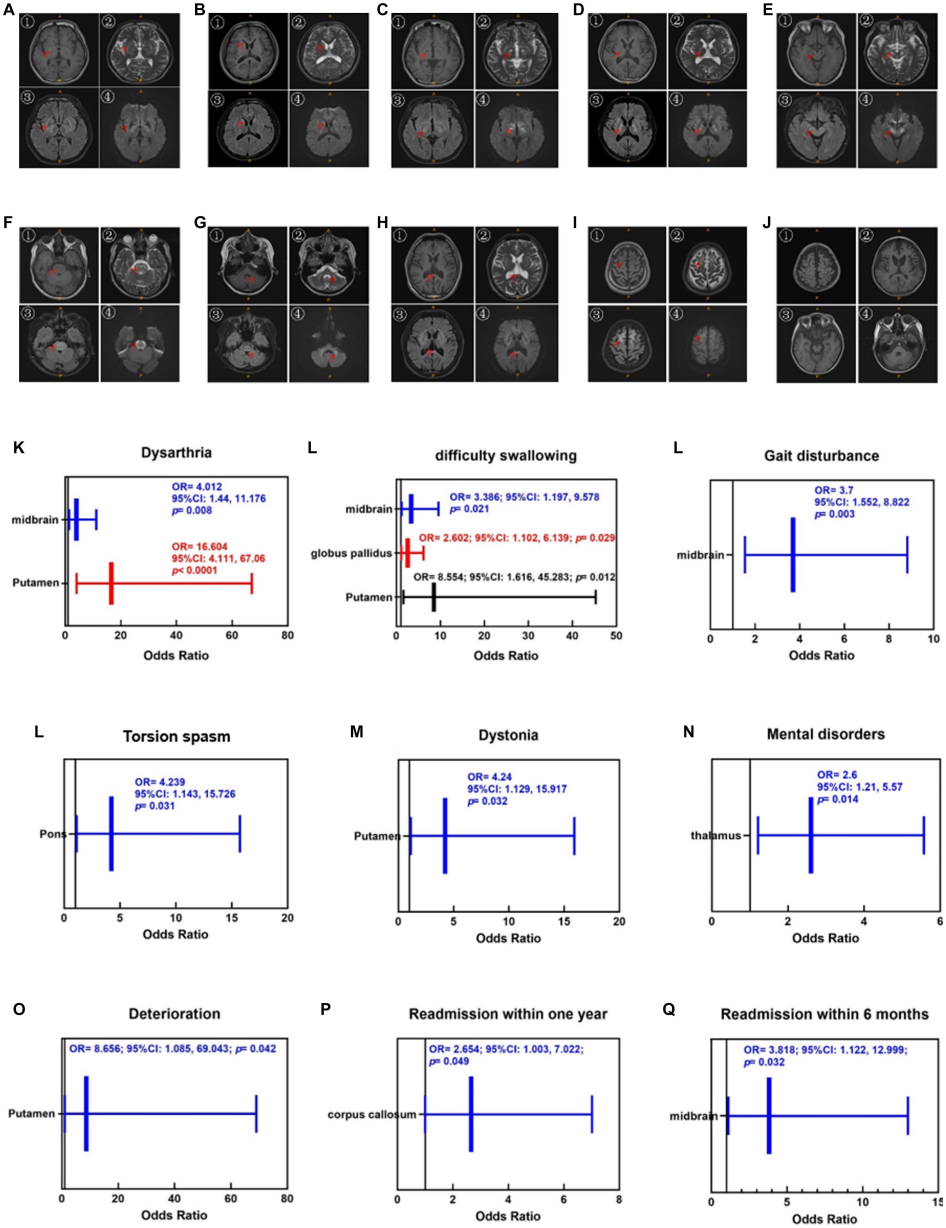
Injuries of various parts of the brain in the T1 (①), T2 (②), Flair (③) and DWI (④) sequences. **(A)** Putamen and globus pallidus, **(B)** caudate nucleus, **(C)** thalamus, **(D)** inner capsule, **(E)** midbrain, **(F)** pons, **(G)** cerebellum, **(H)** corpus callosum, **(I)** cortex, **(J)** brain atrophy. The red arrows mark the specific site of the injury. Brain atrophy can only be observed in the T1 sequence. Enlargement of the ventricular system, reduction of brainstem volume, widening of the tricorn and cortical sulcus fissure can be seen when brain atrophy occurs. **(K–N)** Correlations between cranial injury site and symptoms. **(O–Q)** Correlations between cranial injury site and therapeutic regimen.

Finally, the average scores of the two evaluations were calculated. To avoid subjective bias, two researchers independently evaluated the signal abnormality of all images. The specific scoring rules are listed (Table 1). Figures 2A–J shows the injuries to various parts of the brain in the T1, T2, FLAIR, and DWI sequences.

**Table 1:** Cranial injury score of patients with WD.

Site of injury	T1	T2	FLAIR	DWI
**Original**				
Putamen	1	1	1	2
Globus pallidus	1	1	1	2
Caudate nucleus head	1	1	1	2
Inner capsule	1	1	1	2
Thalamus	1	1	1	2
Midbrain	1	1	1	2
Pons	1	1	1	2
Medulla oblongata	1	1	1	2
Cerebellum	1	1	1	2
Cortex	1	1	1	2
Corpus callosum	1	1	1	2
Brain atrophy	1			
Total score				
**Optimized**				
Lentiform nucleus	1	1	1	18
Caudate nucleus head	1	1	1	2
Thalamus	1	1	1	2
Midbrain	1	1	1	4
Pons	1	1	1	2
Cerebellum	1	1	1	2
Corpus callosum	1	1	1	6
Brain atrophy	1			
Score				

Flair, fluid-attenuation inversion recovery; DWI, diffusion weighted imaging. The degree of injury was not taken into account, and points were scored whenever there was damage in a specific area.

### 2.4. Statistical analysis

Statistical analyses were performed with R (Version 4.2.1 for Windows, Comprehensive R Archive Network).^1^ To determine agreement between two scorers, the intraclass correlation coefficient (ICC) was calculated to determine the inter-rater reliability. A Student’s *t*-test of two independent samples was used for normally distributed data, and the Mann–Whitney U test was used for non-normally distributed data. Data were analyzed using the Chi-square test. The Pearson or Spearman correlation test was used for the correlation analysis. The univariate analysis used the logistic regression model selection method, and statistical significance was set at *p* < 0.05.

## 3. Results

### 3.1. General characteristics of the patients with WD

The enrolled patients were divided into two groups according to whether they were newly diagnosed. No significant differences (*p* > 0.05) were found in terms of sex, subtype, UWDRS scale, ADL score, highest urine copper level before treatment, serum copper level, ceruloplasmin level, and imaging scores between the newly diagnosed group (*n* = 22) and referral group (*n* = 96). However, the age at onset was higher in the newly diagnosed group than in the referral group (*p* < 0.05). Detailed data are presented in Table 2, Part I.

**Table 2:** General information and injury sites of patient with WD in the newly diagnosed group and the referral group.

	the referral group (*n* = 96)	the newly diagnosed group (*n* = 22)	*χ*^2^, *Z*	*p*
Sex (male/female)	63/33	14/8	0.031	>0.05
Hepatic subtype/neurologic subtype	10/86	4/18	0.937	0.293
Age of onset	15 (10, 19)	19.91 ± 7.61	−2.37	**0.018**
UWDRS-I	45.5 (36, 60.75)	66.55 ± 47.99	−1.185	0.236
UWDRS-II	6 (4, 7)	5 (5, 8)	−0.708	0.479
UWDRS-III	6.5 (5, 9)	6.5 (5, 8.75)	−0.421	0.674
UWDRS	58 (46.25, 74.75)	82.32 ± 48.48	−1.579	0.114
ADL (able/disabled)	85/11	16/6	3.63	0.087
Urine copper before treatment	194.97 (120.34, 288.37)	274.12 ± 165.32	−1.106	0.269
Highest urine copper	1324.96 (785.45, 2066.23)	1199.73 ± 469.39	−1.258	0.209
Serum copper	2.71 (1.65, 4.05)	2.12 (1.19, 5.32)	−0.308	0.758
ceruloplasmin	45.15 (36.25, 61.23)	47.95 (33.63, 67.45)	−0.373	0.709
Total score of imaging	9.5 (7, 14)	14.32 ± 9.14	−1.741	0.082
Deterioration (yes/no)	38/58	7/15	0.457	0.628
Whether to be readmitted to hospital within 6 months (yes/no)	19/77	9/13	4.41	0.051
Whether to be readmitted to hospital within a year (yes/no)	38/58	16/6	7.922	**0.008**
Putamen (yes/no)	81/11	18/4	0.673	0.476
globus pallidus (yes/no)	38/58	10/12	0.256	0.637
caudate nucleus (yes/no)	26/70	8/14	0.752	0.437
Inner capsule (yes/no)	6/90	1/21	0.099	1
Thalamus (yes/no)	39/57	11/11	0.644	0.478
Midbrain (yes/no)	64/32	17/5	0.98	0.447
Pons (yes/no)	51/45	16/6	2.802	0.103
cerebellum (yes/no)	38/58	10/12	0.256	0.637
medulla (yes/no)	1/95	0/22	0.415	1
cortex (yes/no)	17/79	5/17	0.286	0.556
corpus callosum (yes/no)	17/79	2/20	1.102	0.521
Brain atrophy (yes/no)	86/10	20/2	0.035	1
Putamen DWI hyper-intensity (yes/no)	7/89	6/16	7.289	**0.015**
globus pallidus DWI hyper-intensity (yes/no)	1/95	1/21	1.022	0.339
caudate nucleus DWI hyper-intensity (yes/no)	2/94	3/19	4.429	**0.044**
Inner capsule DWI hyper-intensity (yes/no)	1/95	1/21	1.022	0.339
Thalamus DWI hyper-intensity (yes/no)	5/91	6/16	8.077	**0.005**
Midbrain DWI hyper-intensity (yes/no)	6/90	6/16	8.659	**0.009**
Pons DWI hyper-intensity (yes/no)	2/94	4/18	7.132	**0.011**
Cerebellum DWI hyper-intensity (yes/no)	2/94	4/18	7.132	**0.011**
Medulla DWI hyper-intensity (yes/no)	1/95	0/22	0.415	1
Cortex DWI hyper-intensity (yes/no)	1/95	1/21	1.022	0.339
corpus callosum DWI hyper-intensity (yes/no)	23/73	2/20	2.755	0.156

The significant difference was marked with bold.

### 3.2. Difference in symptoms between newly diagnosed patients and patients referred with WD

The differences in symptoms between patients in the newly diagnosed and referral groups. Compared to the newly diagnosed group, choreoathetosis and torsion spasms were significantly lower in the referral group (*p* < 0.05), whereas no significant differences were found in terms of dysarthria, difficulty swallowing, tremor, gait disturbance, salivation, dystonia, bradykinesia and rigidity, ataxia, mental disorders, and epilepsy (*p* > 0.05).

### 3.3. Analysis of injury sites between newly diagnosed patients and patients referred with WD

Table 2, Part II shows the injury sites of the newly diagnosed and referred patients with WD. The putamen, globus pallidus, caudate nucleus, thalamus, midbrain, pons, and cerebellum are the main damage sites for copper accumulation in patients with WD and brain atrophy. Damage to the cortex and corpus callosum is rare, and medullary damage has only been found in a single patient. There was no significant difference in the common sites between the two groups, as observed on MRI (*p* > 0.05).

### 3.4. Correlations between the cranial injury site and symptoms

As shown in Figures 2K–N, the correlations between the cranial injury site and clinical symptoms. Putamen injuries were strongly associated with dysarthria (OR = 16.604, 95%CI [4.111, 67.06], *p* < 0.001), dysphagia (OR = 8.554, 95%CI [1.616, 45.283], *p* < 0.05), and dystonia (OR = 4.24, 95%CI [1.129, 15.917], *p* < 0.05). Midbrain injury was strongly associated with dysarthria (OR = 4.012, 95%CI [1.44, 11.176], *p* < 0.01), dysphagia (OR = 3.386, 95%CI [1.197, 9.578], *p* < 0.05), and gait disturbances (OR = 3.7, 95%CI [1.552, 8.822], *p* < 0.01). Pontine injuries were correlated with torsion spasm (OR = 4.239, 95%CI [1.143, 15.726], *p* < 0.05). Thalamic injuries were associated with mental disorders (OR = 2.6, 95%CI [1.124, 5.568], *p* < 0.05).

### 3.5. Risk prediction of DWI hyperintensity at different sites for deterioration or not during treatment

In this study, we included the DWI sequence as an evaluation index because DWI hyperintensity usually represents acute damage. Therefore, we increased the score weight by up to eight times in the case of DWI hyperintensities. The proportion of hyperintensity in the nucleus, caudate nucleus, thalamus, pons, and cerebellum in the newly diagnosed patient group was significantly higher than in the referral patient group. In contrast, DWI hyperintensity in the globus pallidus, internal capsule, medulla, and cortex in the two groups was rare, and the difference was not statistically significant. Patients in both groups were likely to have DWI hyperintensities in the midbrain and corpus callosum, but the difference was not statistically significant.

Therefore, we substituted the putamen, caudate nucleus, thalamus, pons, cerebellum, midbrain, and corpus callosum as independent variables into the logistic regression equation, and OR were calculated using a backward method by taking “whether it was aggravated during treatment,” “whether it was readmitted to the hospital within 6 months,” and “whether it was readmitted within 1 year” as a dependent variable, respectively. As shown in Figures 2O–Q, putamen injury was strongly associated with deterioration during treatment (OR = 8.656, 95%CI [1.085, 69.043], *p* < 0.05), corpus callosum injury was strongly associated with readmission within 1 year (OR = 2.654, 95%CI [1.003, 7.022], *p* < 0.05), and midbrain injury was strongly associated with readmission within 6 months (OR = 3.818, 95%CI [1.122, 12.999], *p* < 0.05).

### 3.6. Consensus weighting of the semi-quantitative scoring system for cranial MR in WD

Intrarater agreement (ICC) was good for two scorers, that is for MRI score (0.855 [0.798–0.897], *p* < 0.0001).

According to the results detailed in 3.3 and 3.5, we found that DWI hyperintensities in the lentiform nucleus (putamen and globus pallidus) and midbrain were strongly associated with prognosis and brain atrophy. Therefore, the integral weights of the DWI hyperintensities of the putamen, midbrain, and corpus callosum should be increased to prove the susceptibility of the semi-quantitative scoring system for clinical prediction. Here, we provide an consensus algorithm as presented in Table 1. In this new scoring system, the point weights of the caudate nucleus, thalamus, pons, and cerebellum in the sequences of T1, T2, FLAIR, and DWI remained unchanged compared with the former version (Table 1). The weights of the DWI hyperintensity in the lentiform nucleus, midbrain, and corpus callosum increased to 18, 4, and 6, respectively.

**Figure 3:**
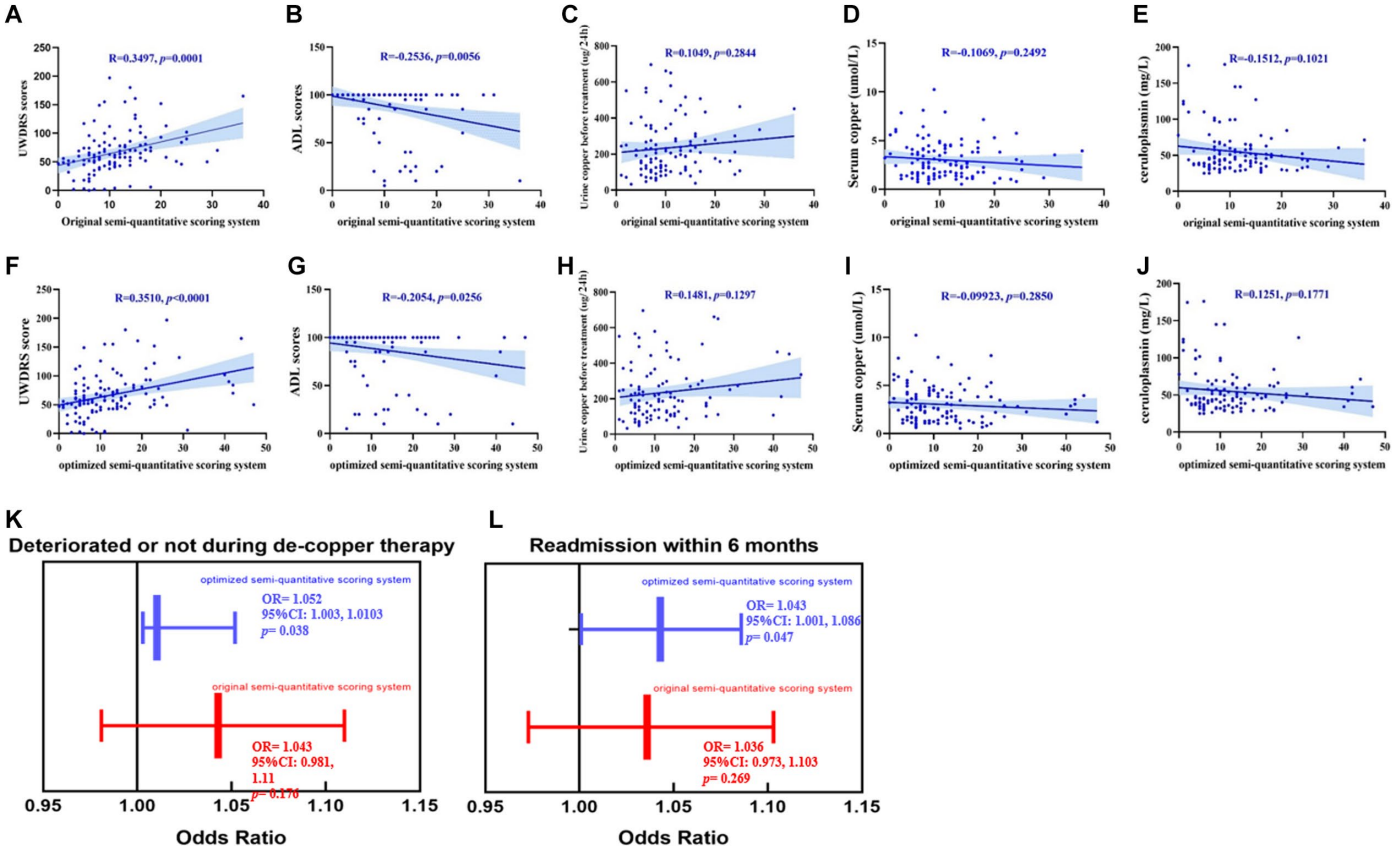
**(A–J)** The promotion of the consensus semi-quantitative scoring system compared to the original semi-quantitative scoring system, both their correlations between the UWDRS score, ADL score and other clinical indexes (urine copper before treatment, serum copper and ceruloplasmin). **(K,L)** The consensus imaging score had a strong correlation between deterioration during treatment [OR = 1.052, 95%CI (1.003, 1.0103), *p* < 0.05] and readmission within 6 months [OR = 1.043, 95%CI (1.001, 1.086), *p* < 0.05]. While no significant correlations had been found between the original imaging score and deterioration during treatment [OR = 1.043, 95%CI (0.981, 1.11), *p* > 0.05] and readmission within 6 months [OR = 1.036, 95%CI (0.973, 1.103), *p* > 0.05].

As shown in Figures 3A–J, the original and consensus semi-quantitative scoring systems showed strong correlations between the UWDRS and ADL scores. The positive correlation of UWDRS with the consensus semi-quantitative scoring system (*r* = 0.351, *p* < 0.001) and original semi-quantitative scoring system (*r* = 0.35, *p* < 0.001), while it was both negative correlations to the ADL score (consensus semi-quantitative scoring system, *r* = −0.205, *p* < 0.05; original semi-quantitative scoring system, *r* = −0.254, *p* < 0.01). No significant differences were found between the two systems and other clinical indices (urine copper before treatment, serum copper, and ceruloplasmin).

As shown in Figures 3K,L, we reassessed the predictive performance of the weighted cranial DWI scale. Compared with the original imaging score, the weighted imaging score had a similar result with deterioration during treatment (OR = 1.052, 95%CI [1.003, 1.0103], *p* < 0.05) and readmission within 6 months (OR = 1.043, 95%CI [1.001, 1.086], *p* < 0.05).

## 4. Discussion

In the early stages of WD treatment, there may be a deterioration of neurological symptoms, which could be associated with copper-related oxidative stress reactions (Ziemssen et al., 2022). However, the current approach to preventing the deterioration of neurological symptoms during treatment is mostly based on physicians’ experience, rather than reliable clinical markers (Hou et al., 2022). According to our results, there was no significant difference in terms of gender composition, clinical phenotypes, UWDRS scale scores, cranial MRI imaging scores, 24-h urinary copper, ceruloplasmin, serum copper and ADL scores between newly diagnosed patients and referral patients. This indicated the limited specificity of clinical assessment methods in evaluating the efficacy and prognosis of WD patients, emphasizing the need for more reliable clinical markers (Hou et al., 2022). The imaging scoring system developed by Dusek et al. (2020) can reflect the alleviation of acute cranial brain injury in WD patients as treatment progresses, which indicated that neuroimaging may serve as a potential reliable tool for prognostic prediction. Considering the mild changes in chronic injuries over the short term and the focus trend of damage in the regions of interest, we adjusted the scoring strategy for assessing cranial MRI damage in WD according to former research. Adjustments include increasing of weight coefficient of DWI hyperintensity and specific regions of interest. Subsequently, we verified the clinical value of this scoring system by establishing the regression relationships between the imaging scores and clinical outcomes. Including deterioration during de-copper therapy and readmission within 6/12 months.

DWI hyperintensity representing acute cranial brain injuries may exhibit greater predictive value and potential for assessing treatment efficacy in the short term, compared to chronic injuries (Chen et al., 2017). In this study, no significant differences had been found in the occurrence rates of neurological symptoms related to brain damage, such as dysarthria, dysphagia, tremor, gait disturbances, sialorrhea, dystonia, bradykinesia, muscular rigidity, ataxia, psychiatric disorders, and epilepsy, between newly diagnosed patients and referral patients. These findings suggest that cranial abnormalities in patients with neurologic WD are correlated with their clinical symptoms, age of onset, and disease course (Yu et al., 2019). Previous studies have indicated that common manifestations on cranial MRI lesions of patients with WD include: T1 hypointensity/T2 hyperintensity, T1 hyperintensity and T1/T2 hypointensity, which may be due to the copper accumulation in different disease courses and different brain regions (Li et al., 2019). Sener (2003) found that the restricted water diffusion presenting as DWI hyperintensity/ADC hypointensity is due to cellular inflammatory injury and toxic edema caused by copper deposition among patients with WD (Pulai et al., 2014). In newly diagnosed patients with WD, there is a relatively high proportion of DWI hyperintensity in the caudate nucleus, putamen, thalamus, midbrain, pons, and cerebellum. When considering the apparent diffusion coefficient (ADC) sequence to determine whether DWI hyperintensity represents water diffusion, we found the DWI hyperintensity was caused by T2 shine-through effect in some patients. Therefore, simultaneous presence of DWI hyperintensity and ADC hypointensity was considered as the criterion for abnormal DWI intensity, to exclude the interference of T2 shine-through effect. In conclusion, based on the higher specificity and clinical significance, we have assigned a greater weight to DWI hyperintensity as an important scoring criterion in our assessment.

There is a correlation between different clinical presentations and the location of cranial MRI lesions among patients with WD (Yu et al., 2019). Previously, it was believed that a specific clinical symptom in patients with WD is likely caused by simultaneous damage from different brain regions rather than being directly attributed to the injury of a single brain region (Zhong et al., 2019). In this study, to adjust the weight coefficients, we calculated the OR values of abnormal MRI intensity in different brain regions corresponding to clinical symptoms. Occurrence of putamen injuries promoted the risk of dysarthria, dysphagia and dystonia by 16.604, 8.554 and 4.24 times, respectively. Occurrence of midbrain injuries promoted the risk of dysarthria, dysphagia and gait disturbances by 4.012, 3.386 and 3.7 times, respectively. Occurrence of pontine injuries promoted the risk of torsion spasm by 4.239 times. The occurrence of thalamic injuries promoted the risk of mental disorders by 2.6 times. Additionally, patients with DWI hyperintensity in the putamen were more prone to experiencing deterioration of neurological symptoms during the de-copper treatment. Patients with DWI hyperintensity in the corpus callosum and midbrain may require de-copper therapy within 6/12 months to stabilize the condition.

Sinha et al. (2006) and Selwa et al. (1993) graded quantification criteria for cranial injury in patients with WD, grade was done for the severity of change in signal intensity (deviation from the conventionally accepted signal intensity of a given structure in a particular sequence) and associated atrophy (0 = no abnormality, 1 = change in signal intensity with no atrophy, 2 = change in signal intensity with mild or moderate atrophy, and 3 = change in signal intensity with severe atrophy). MRI changes correlated with the mean NSS, S and E activities of daily living, and Chu staging scores (*p* < 0.001), but did not correlate with the duration of illness. Sinha et al. (2007) investigated the changes in cranial MRI and clinical symptoms in 50 patients with WD, revealing varying degrees of improvement in both MRI and clinical manifestations following treatment. They also found a strong correlation between pre-and post-treatment Neurological Severity Score (NSS) and cranial MRI scores, indicating that both can serve as indicators of treatment effectiveness in WD patients. Their results demonstrate that this method is an effective means of observing treatment response in WD patients. Li (2019) employed the grading method proposed by Sinha et al. (2006) to quantify abnormal signals in WD cranial MRI. This method, based on the T2 sequence, categorizes abnormal signals into hyperintensity and hypointensity and assigns them into four grades based on lesion size: no abnormality = 0 points, mild = 1 point, moderate = 2 points, severe = 3 points. This method can reflect the extent of copper deposition and neural damage in the brains of WD patients. However, it does not provide information regarding the changes and significance of this method during copper chelation therapy. Dusek et al. (2020) developed a semi-quantitative method for assessing abnormalities in WD cranial MRI, which is divided into acute and chronic parts. The acute toxicity scores ranged from 0 to 2 based on the degree of T2/FLAIR hyperintensity in the caudate nucleus, putamen, thalamus, midbrain, and pons. The chronic damage scores were based on the presence of cerebral atrophy and T2/SWI hypointensity. This method demonstrated high consistency and effectiveness in a 24-month follow-up of 37 patients. Changes in MRI scores were primarily driven by changes in the acute and chronic scores. The MRI scores were positively correlated with the UWDRS-III psychiatric scale, indicating that this method can semi-quantitatively reflect the severity of WD. In this study, we explored an improved scoring method for cranial MRI injuries, aiming to assess the extent of brain damage and treatment effectiveness in patients with WD. Based on previous research, we modified the scoring method for WD cranial MRI injuries by incorporating DWI sequences and assigning them higher scores. Eight brain regions were selected as scoring areas, including the caudate nucleus, putamen, thalamus, midbrain, pons, cerebellum, corpus callosum, and cerebral atrophy. We increased the scores for the caudate nucleus, midbrain, and corpus callosum to emphasize their importance based on the therapeutic implications of DWI hyperintensity in different regions. We analyzed the advantages and disadvantages of the two scoring methods and found that the revised scoring system, which includes reselected scoring areas and scores, can be used to evaluate whether WD patients experience deterioration of neurological symptoms during de-copper therapy and whether they require readmission within 6 months to stabilize the copper homeostasis. Our results indicated that for every one-point increase in cranial MRI scores, there is a 5.2% increased risk of deterioration during treatment and a 4.3% increased risk of readmission within 6 months. We believe that this cranial MRI injury scoring method holds significant clinical implications for guiding the management of WD.

Brain injuries in WD primarily involve multiple brain regions, such as the caudate nucleus, putamen, thalamus, midbrain, pons, cerebellum, and corpus callosum, accompanied by varying degrees of cerebral atrophy (Yu et al., 2019). Currently, MRI examination is one of the crucial tools for cranial injuries assessment (Shribman et al., 2022). However, due to significant inter-individual variations in clinical and radiological manifestations of WD, there is a lack of unified standards and methods for its classification and grading. To solve this issue, this study proposed a weighted-multiple-brain-regions-based approach that extracts quantitative features from multimodal images of WD patients. By combining regression models, we have established a cranial imaging scoring system capable of predicting the prognosis of WD patients. This scoring system demonstrates excellent predictive ability and provides a more objective and effective basis for the clinical treatment of WD patients. The scoring system integrates the key injured brain regions, DWI hyperintensity and clinical therapeutic experience to derive a weighted score that reflects neurological symptoms. We conducted correlation analysis between this score and clinical indicators as well as prognostic factors, revealing its effective discrimination ability among patients with different risk levels and significant associations with treatment response and risk of readmission. The method possesses the advantages of objectivity and simplicity, providing a novel tool for prognostic management of WD patients. Our study demonstrates that this imaging scale exhibits favorable predictive performance for the short-term prognosis of WD patients. However, its long-term performance still requires validation. Only 20.33% (24/118) of patients underwent neuroimaging follow-up, and only 37.50% (9/24) of those patients exhibited changes in neuroimaging scores. This suggests that neuroimaging changes may be stable in the short term. Therefore, in future research, it is necessary to include a larger cohort of follow-up and initial visit patients and attempt to construct clinical prediction models for their treatment by incorporating laboratory indicators such as 24-h urinary copper and serum non-ceruloplasmin-bound copper during therapy. This will enable us to more accurately assess the condition and prognosis of WD patients, providing them with consensus individualized treatment plans.

## Data availability statement

The raw data supporting the conclusions of this article will be made available by the authors, without undue reservation.

## Ethics statement

The studies involving human participants were reviewed and approved by the Ethics Committee of Hefei Institutes of Physical Science, Chinese Academy of Sciences. Written informed consent to participate in this study was provided by the participants’ legal guardian/next of kin. Written informed consent was obtained from the individual(s), and minor(s)’ legal guardian/next of kin, for the publication of any potentially identifiable images or data included in this article.

## Author contributions

S-jW and HG: research project: conception, organization, execution, statistical analysis: design, execution, review, and critique, manuscript preparation: writing the first draft. S-rC: research project: organization, execution, statistical analysis: execution, review, and critique, manuscript preparation: writing the first draft. C-cX and TW: research project: execution, statistical analysis: execution, manuscript preparation: review and critique. YW and BL: research project: organization, statistical analysis: review, and critique, manuscript preparation: review and critique. R-qZ and Y-sH: research project: execution, statistical analysis: review, and critique, manuscript preparation: review and critique. TW, Z-hD, XW, and Y-zH: research project: conception, statistical analysis: review, and critique, manuscript preparation: review and critique. Y-nS and NC: research project: conception, statistical analysis: design, manuscript preparation: review and critique. All authors contributed to the article and approved the submitted version.

## Funding

S-jW received funding from the Natural Science Foundation of Anhui Province (grant number: 2208085QH262) and the Anhui Provincial Department of Education (grant number KJ2021A0552). C-cX received funding from the Natural Science Foundation of China (grant number: 81904086). Z-hD received funding from major projects of Anhui Science and Technology (grant number: 202103a07020004). BL received funding from the Natural Science Research Project of the Anhui Educational Committee (grant number: KJ2021A0564).

## Conflict of interest

The authors declare that the research was conducted in the absence of any commercial or financial relationships that could be construed as a potential conflict of interest.

## Publisher’s note

All claims expressed in this article are solely those of the authors and do not necessarily represent those of their affiliated organizations, or those of the publisher, the editors and the reviewers. Any product that may be evaluated in this article, or claim that may be made by its manufacturer, is not guaranteed or endorsed by the publisher.

## Footnotes

^1^ https://www.r-project.org
